# To: Outcomes of critically ill pregnant COVID-19 patients: a cohort study

**DOI:** 10.62675/2965-2774.20240122-en

**Published:** 2024-10-11

**Authors:** Josef Finsterer

**Affiliations:** 1 Neurology and Neurophysiology Center Vienna Austria Neurology and Neurophysiology Center - Vienna, Austria,

To the Editor

We read with interest the article by Soares et al. on a retrospective, observational, single-center study of the outcomes of critically ill pregnant women with coronavirus disease 2019 (COVID-19) pneumonia (n = 16) requiring intensive care unit (ICU) admission compared with nonpregnant women with COVID-19 pneumonia requiring ICU admission (n = 746). Thirteen of the 16 pregnant women required mechanical ventilation. The mortality rate was 25%, and the number of ventilator-free days (VFDs) ranged from 5 - 15 days in pregnant women and 0 - 20 days in controls on day 28. Mortality and the number of VFDs did not differ between the two groups. The study is impressive, but some points require further discussion.^(1)^

The first limitation of the study is the retrospective design. Retrospective designs have the disadvantage that some data may be missing, the accuracy of the data cannot be easily checked, desired missing or new data cannot be generated, and recommendations for specific investigations are often not comprehensible. A retrospective design also does not allow for follow-up studies. The number of people who were excluded due to missing data and to what extent this affected the results should be reported.

The second limitation is that the number of VFDs at day 28 and day 60 were calculated and compared between the two groups, but this number only ranged from 5 - 15 VFDs in the group of pregnant women, and the ICU length of stay was only 10.8 - 23.3 days. How can VFDs at days 28 and 60 be compared between groups if none of the pregnant females remain in the ICU for more than 23 days? This discrepancy should be resolved.

The third limitation is that nine of the 13 ventilated pregnant women developed ventilator-associated pneumonia.^(1)^ Since all 16 patients were admitted to the ICU due to COVID-19, how to differentiate COVID-19 pneumonia from ventilator-associated pneumonia should be reported. Did all nine patients develop ventilator-associated pneumonia after COVID-19 pneumonia subsided?

The fourth limitation is that no information about vaccination status was provided. Since the observational period lasted until May 2021, it is conceivable that at least some pregnant women and controls were already vaccinated. Therefore, how many of the included subjects were vaccinated and did the disease course or outcome differ between vaccinated and unvaccinated subjects should be reported.

The fifth limitation is the small number of included patients. Ventilatory parameters were compared between only 12 survivors and 4 nonsurvivors, making the statistical comparison unreliable. The statistical comparison between controls (n = 746) and pregnant women (n = 16) can also be unreliable due to unequal group sizes and an inhomogeneous cohort structure, which makes statistical comparison problematic.

The sixth limitation is that only 13 of the 16 pregnant women were ventilated. Whether the number of VFDs was calculated for all 16 or only the 13 ventilated patients should be reported.

The seventh limitation is that neurological causes of respiratory failure have not been sufficiently ruled out. Since neurological complications often occur with severe acute respiratory syndrome coronavirus 2 (SARS-CoV-2) infections, central nervous system disease (e.g., brainstem infarction, venous sinus thrombosis brainstem immune encephalitis) and peripheral nervous system disease (e.g., Guillain–Barré syndrome) are completely excluded.

The causes of death of the four pregnant women who died, how many of their newborns survived, and how many of the newborns of the 5 patients whose pregnancies were terminated survived should also be reported. The mortality data and the number of ventilated subjects in the control group were missing. One limitation is that medication use before and during hospitalization was not included in the analysis, and why all enrolled patients received dexamethasone should be explained.

We disagree with the statement in the introduction that there are limited published data on pregnant women with COVID-19.^(1)^ When searching PubMed via the search terms "pregnancy", "pregnant", "SARS-CoV-2 infection", and "COVID-19", we received > 5000 hits.

In summary, this interesting study has limitations that put the results and their interpretation into perspective. Addressing these limitations could strengthen the conclusions and reinforce the study's message. Before respiratory failure in patients with COVID-19 can be attributed solely to viral infection, alternative causes must be ruled out.
