# Supplementary material for: To: Outcomes of critically ill pregnant COVID-19 patients: a cohort study
Source: Crit Care Sci. 2024 Oct 11;36:e20240122en. doi: 10.62675/2965-2774.20240122-en (PMC11554299; doi:10.62675/2965-2774.20240122-en)
Supplement: Translation

# Translation: Para: Desfechos de pacientes gestantes gravemente enfermas com COVID-19: um estudo de coorte

**DOI:** 10.62675/2965-2774.20240122-pt

**Authors:** Josef Finsterer

**Affiliations:** 1 Neurology and Neurophysiology Center Viena Áustria Neurology and Neurophysiology Center - Viena, Áustria

Ao Editor

Lemos com interesse o artigo de Soares et al. sobre um estudo retrospectivo, observacional e unicêntrico dos desfechos de gestantes em estado crítico com pneumonia relacionada à doença causada pelo coronavírus 2019 (COVID-19) (n = 16) que necessitaram de internação em unidade de terapia intensiva (UTI), em comparação com não gestantes com pneumonia por COVID-19 que necessitaram de internação em UTI (n = 746). Das 16 gestantes, 13 necessitaram de ventilação mecânica. A taxa de mortalidade foi de 25%, e o número de dias sem ventilador (DSVs) variou de 5 a 15 nas gestantes e de zero a 20 no grupo controle em 28 dias. A mortalidade e o número de DSVs não diferiram entre os dois grupos. O estudo é notável, mas alguns pontos precisam ser discutidos.^(1)^

A primeira limitação do estudo é o desenho retrospectivo, o qual têm a desvantagem de que alguns dados podem estar faltando, a precisão dos dados não pode ser facilmente verificada, os dados desejados faltantes ou os novos dados não podem ser gerados e geralmente não são compreensíveis as recomendações de investigações específicas. O desenho retrospectivo também não permite estudos de seguimento. Deve-se relatar o número de indivíduos excluídos devido à falta de dados e até que ponto isso afetou os resultados.

A segunda limitação é que o número de DSVs em 28 e 60 dias foi calculado e comparado entre os dois grupos, mas esse número variou de 5 a 15 DSVs no grupo das gestantes, e o tempo de internação na UTI foi de apenas 10,8 a 23,3 dias. Como é possível comparar os DSVs nos dias 28 e 60 entre os grupos se nenhuma das gestantes permanece na UTI por mais de 23 dias? Deve-se resolver essa discrepância.

A terceira limitação é que 9 das 13 gestantes ventiladas desenvolveram pneumonia associada à ventilação mecânica.^(1)^ Como todas as 16 pacientes foram admitidas na UTI devido à COVID-19, é preciso informar como diferenciar a pneumonia por COVID-19 da pneumonia associada à ventilação mecânica. Todas as nove pacientes desenvolveram pneumonia associada ao ventilador depois que a pneumonia por COVID-19 foi controlada?

A quarta limitação é que não foram fornecidas informações sobre o esquema de vacinação. Como o período de observação durou até maio de 2021, é possível que pelo menos algumas gestantes e controles já estivessem imunizadas. Portanto, é preciso informar quantos dos pacientes incluídos foram vacinados e se o curso ou o desfecho da doença diferiu entre os indivíduos vacinados e os não vacinados.

A quinta limitação é o número reduzido de pacientes incluídas. Os parâmetros ventilatórios foram comparados entre apenas 12 sobreviventes e 4 não sobreviventes, tornando a comparação estatística não confiável. A comparação estatística entre controles (n = 746) e gestantes (n = 16) também pode não ser confiável devido ao tamanho desigual dos grupos e a uma estrutura de coorte não homogênea, o que torna difícil a comparação estatística.

A sexta limitação é que apenas 13 das 16 gestantes foram ventiladas. Deve-se informar se foi calculado o número de DSVs para todas as 16 ou somente para as 13 gestantes ventiladas.

A sétima limitação é que as causas neurológicas da insuficiência respiratória não foram suficientemente descartadas. Como as complicações neurológicas ocorrem com frequência em infecções por coronavírus da síndrome respiratória aguda grave 2 (SARS-CoV-2), foram totalmente excluídas doenças do sistema nervoso central (por exemplo: infarto do tronco cerebral, trombose do seio venoso, encefalite imune do tronco cerebral) e doenças do sistema nervoso periférico (por exemplo: síndrome de Guillain-Barré).

As causas de morte das quatro gestantes, quantos de seus recém-nascidos sobreviveram e quantos dos recém-nascidos das cinco pacientes cujas gestações foram interrompidas sobreviveram também devem ser relatadas. Faltaram dados de mortalidade e o número de indivíduos ventilados no grupo controle. Uma limitação é que o uso de medicamentos antes e durante a hospitalização não foi incluído na análise, e deve-se explicar o motivo pelo qual todos os pacientes incluídos receberam dexametasona.

Discordamos da afirmação na introdução de que há poucos dados publicados de gestantes com COVID-19.^(1)^ Ao pesquisar no PubMed com os termos de busca "pregnancy", "pregnant", "SARS-CoV-2 infection" e "COVID-19", obtivemos mais de 5.000 resultados.

Em resumo, esse interessante estudo tem limitações que colocam os resultados e sua interpretação em perspectiva. A abordagem dessas limitações poderia fortalecer as conclusões e reforçar a mensagem do estudo. Antes que a insuficiência respiratória em pacientes com COVID-19 possa ser atribuída exclusivamente à infecção viral, devem-se descartar causas alternativas.
